# Antioxidant Capacity, Antitumor Activity and Metabolomic Profile of a Beetroot Peel Flour

**DOI:** 10.3390/metabo13020277

**Published:** 2023-02-14

**Authors:** Pedro Paulo Saldanha Coimbra, Anna Carolina Alves Gomes da Silva-e-Silva, Ananda da Silva Antonio, Henrique Marcelo Gualberto Pereira, Valdir Florêncio da Veiga-Junior, Israel Felzenszwalb, Carlos Fernando Araujo-Lima, Anderson Junger Teodoro

**Affiliations:** 1 Food and Nutrition Graduate Program, Federal University of Rio de Janeiro State, Rio de Janeiro 21941-901, Brazil; 2 Laboratory of Environmental Mutagenicity, Department of Biophysics and Biometry, Rio de Janeiro State University, Rio de Janeiro 20550-013, Brazil; 3 Laboratory for the Support of Technological Development, Chemistry Institute, Federal University of Rio de Janeiro, Rio de Janeiro 21941-901, Brazil; 4 Department of Chemical Engineering, Military Institute of Engineering—IME, Rio de Janeiro 22290-270, Brazil; 5 Department of Genetics and Molecular Biology, Federal University of Rio de Janeiro State, Rio de Janeiro 21941-901, Brazil; 6 Department of Nutrition and Dietetics, Faculty of Nutrition, Fluminense Federal University, Rio de Janeiro 24020-141, Brazil

**Keywords:** *Beta vulgaris* L., betalains, breast cancer, chemical profiling

## Abstract

In this study, a beetroot peel flour was made, and its in vitro antioxidant activity was determined in aqueous (BPFw) and ethanolic (BPFe) extracts. The influence of BPFw on breast cancer cell viability was also determined. A targeted betalain profile was obtained using high-resolution Q-Extractive Plus Orbitrap mass spectrometry (Obrtitrap-HRMS) alongside untargeted chemical profiling of BPFw using Ultra-High-Performance Liquid Chromatography with High-Resolution Mass Spectrometry (UHPLC-HRMS). BPFw and BPFe presented satisfactory antioxidant activities, with emphasis on the total phenolic compounds and ORAC results for BPFw (301.64 ± 0.20 mg GAE/100 g and 3032.78 ± 55.00 µmol T/100 g, respectively). The MCF-7 and MDA-MB-231 breast cancer cells presented reductions in viability when treated with BPFw, showing dose-dependent behavior, with MDA-MB-231 also showing time-dependent behavior. The chemical profiling of BPFw led to the identification of 9 betalains and 59 other compounds distributed amongst 28 chemical classes, with flavonoids and their derivates and coumarins being the most abundant. Three forms of betalain generated via thermal degradation were identified. However, regardless of thermal processing, the BPF still presented satisfactory antioxidant and anticancer activities, possibly due to synergism with other identified molecules with reported anticancer activities via different metabolic pathways.

## 1. Introduction

Beetroot (*Beta vulgaris* L.) is a vegetable with great commercial and nutritional importance from the *Chenopodiaceae* family; the red-purplish root is consumed in natura, cooked or processed, with the form of consumption varying according to the customs of the people [1].

Beetroot extracts have previously been investigated as a source of bioactive compounds with high antioxidant activity, evaluated by using various in vitro methods with synthetic free radicals, such as 2,2-azinobis (3-ethylbenzothiazoline-6-sulfonic acid (ABTS) and 2,2-diphenyl-picrilhidrazil (DPPH) assays, and by examining the ability to interact with the oxygen and/or peroxides in oxygen radical absorbance capacity (ORAC) assays [2].

The oxidative stress promoted by the aerobic metabolism of a cell is associated with different non-communicable chronic diseases, including cancer, since the damage promoted by reactive species (including oxygen, sulfur, nitrogen and chloride species) depends not only on their concentration and formation but also on the activity and availability of the internal mechanisms of the antioxidant enzymatic system and antioxidant compounds [3,4]. With an excess of reactive species, some relevant cell structures can be degraded, such as cell membrane proteins and enzymes, as well as the DNA, leading to important cell damage and possible mutations, which can promote cell death or an increase in the activity of survival mechanisms. In the second case, the cell can mutate, giving rise to cancer [3].

Cancer is a multifactorial disease, and it is correlated with intrinsic and extrinsic factors that can contribute to its formation, progression and prevention [4]. Cancer is still one of the leading causes of death worldwide, with breast cancer being the main form of cancer affecting women [5].

Alongside the aforementioned in vitro antioxidant activity, beetroot extracts with anticancer activity against lung [6], prostate [2], liver, tongue and colon cancers have also been reported [7]. Extracts have obtained been from different parts of the beetroot plant, such as the flesh [8], leaves [2], roots [9] and peel [10], with betalains and polyphenols receiving great attention. Betalains have previously been reported not only as antioxidants [11] but also as molecular agents that act via different pathways, such as via the inhibition of the mTOR pathway, the positive modulation of the caspases pathway [12], and the activation of the p53 pathway [6], leading to the modulation of the cell cycle and apoptosis [13].

The fact that different parts of the beetroot plant can be used for the extraction of bioactive compounds follows a growing interest in the full use of fruits and vegetables, and it shows synergy with the Sustainable Development Goals of the United Nations, particularly with the 12th goal, which refers to responsible production and consumption, including in agriculture [14]. However, the food habits of each population affect the vegetable residues that are produced, and they are difficult to overcome [1]. When it comes to the beetroot plant (*Beta vulgaris* L.), the most commonly consumed part is the flesh, with the roots and leaves also being appreciated in some cultures, leaving the peel as the main residue [15].

In order to promote the use of vegetable residues, the production of vegetable flours is an option, since they can preserve the bioactivity and nutritional properties of the in natura vegetable [16]. Beetroot peel is promising for the production of flour due to its proximate composition, containing a protein content of almost 7%, a dietary fiber content of 19% and a mineral content of 11% (dry weight basis), as well as bioactive compounds, such as polyphenols, flavonoids and betalains [17,18]. However, although the preparation of flours from plant residues has been reported in the literature as a process capable of preserving biological activities, the drying temperature may cause the thermal degradation of bioactive compounds, thus reducing their activity [16]. Therefore, investigating the antioxidant and anticancer activities of flours made from plant residues is very relevant, and it provides a basis for the continued development of plant byproducts.

In addition, identifying compounds that can interact with molecular targets for cancer treatment and/or prevention is essential, since it creates the opportunity to evaluate the extension of the activity and, if necessary, to purify the compound for further use, with different plant sources of antioxidants and other bioactive compounds being investigated [19]. Thus, the objectives of this work were to evaluate whether there are antioxidant and anticancer activities in beetroot peel flour after processing and to identify the presence of anticancer compounds in this flour.

## 2. Materials and Methodology

### 2.1. Flour Development

Beetroot peel (*Beta vulgaris* L.) was acquired from the local market in Rio de Janeiro, Brazil. The peel was maintained in translucent, hermetically closed plastic bags and placed in thermal bags during transport. The peel samples were selected for the removal of inappropriate portions, sanitized with 10% sodium hypochlorite for 15 min, drained, rinsed in water and dried with paper. The sanitized peel was processed in a food processor model OMPR550–127, 127 V, 300 W (Oster^®^, Santa Catarina, Brazil) using 2 cycles of 20 s each, and then it was dried in a food dryer ST-04T, 110 V, 500 W (Colzer^®^, Villeurbanne, France) at 70 °C for 150 min. The dried peel was submitted to two further processing cycles of 20 s each (model OMPR550–127, 127 V, 300 W, Oster^®^, Santa Catarina, Brazil), resulting in beetroot peel flour (BPF).

### 2.2. Antioxidant Activity

The bioactive compounds were extracted from the BPF using ultrapure water or a 50% ethanol solution, both at a ratio of 1:3 (BPF:water; m:v), and they were denominated as BPFw or BPFe, respectively, and stored until analyzed. The extracts were homogenized, three different dilutions were prepared, and readings were carried out in a microplate reader/fluorimeter SpectraMax i3x Multi-Mode Microplate Reader (Molecular Devices, San Jose, CA, USA) for all methods. The details of the wavelengths and plate preparations are provided together with the descriptions of each assay.

#### 2.2.1. Total Phenolic Compounds (TPCs)

The total phenolic compounds were quantified by using the Folin–Ciocalteu method as adapted by Abreu et al. [20]. A volume of 30 µL of each BPF dilution was added to a 96-well plate with 150 µL of a 10% solution of Folin–Ciocalteu reagent. The reaction was allowed to occur for 5 min, and then 120 µL of a 4% solution of calcium carbonate was added. The plate was stored in the dark at ambient temperature, and the reading was made at 750 nm. The results are reported as mg of gallic acid equivalents per 100 g of BPF (mg GAE/100 g).

#### 2.2.2. ABTS Radical Scavenging

An ABTS assay was carried out as described by Rufino et al. [21]. The ABTS solution was prepared by dissolving the synthetic free radical 2,2-azinobis (3-ethylbenzothiazoline-6-sulfonic acid in ultrapure water, and then it was added to a 96-well microplate. The BPF dilutions were added, the microplate was maintained for 6 min at ambient temperature in the dark, and then the reading was made at 734 nm. The results are reported as µM of Trolox per 100 g of BPF (µM T/100 g).

#### 2.2.3. DPPH Radical Scavenging

A DPPH assay was carried out as described by Brand-Williams et al. [22] with modifications. A 2.4 mg% methanolic solution of the 2,2-diphenyl-picrilhidrazil (DPPH) synthetic free radical was added to a 96-well microplate with different aliquots of the BPF dilutions. The readings were carried out at 515 nm, and the results are reported as µM of Trolox per 100 g of BPF (µM T/100 g).

#### 2.2.4. Ferric Iron Reducing Antioxidant Parameter (FRAP)

A FRAP assay was carried out as described by Thaipong et al. [23]. BPF dilutions were added to 96-well microplates together with acetate buffer (0.3 M, pH 3.6), 10 mM TPTZ and iron chloride 20 mM solutions. The microplates were incubated at 37 °C, and the absorbance was determined at 595 nm. The results are given as µM of ferrous sulfate per gram of BPF (µM FS/g).

#### 2.2.5. Oxygen Radical Absorbance Capacity (ORAC)

An ORAC assay was carried out as described by Prior et al. [24]. The excitation wavelength was 485 nm, while the emission wavelength was 520 nm. The results are given as µM of Trolox per 100 g of BPF (µM T/100 g).

### 2.3. Influence on Cancer Cells

#### 2.3.1. BPF Extract Preparation

A 50 mg·mL^−1^ stock solution (BPF:distilled water) was prepared, maintained in a water bath at 37 °C for 30 min and then centrifuged at 1500 RPM for 5 min. The supernatant was recovered and filtered using a syringe filter (0.22 µm pore size, KASVI, PR, Brazil). The filtered extracts were kept frozen until analyzed. For use, the extract was diluted with medium (1:1, *v*:*v*), and then different solutions with concentrations ranging from 2.5 mg to 25 mg/mL of BPF were prepared.

#### 2.3.2. Cell Culture

Breast cancer was studied using the MCF-7 (HTB-22 ATCC) and MDA-MB-231 (HTB-26 ATCC) models, both being human epithelial breast cancer cell lines. The MCF-7 cells were cultured in Dulbecco’s Modified Eagle medium (DMEM) supplemented with 10% fetal bovine serum and 1% penicillin/streptomycin, with pH 7.4. The MDA-MB-231 cells were cultured in Roswell Park Memorial Institute medium (RPMI 1640) supplemented with 10% fetal bovine serum and 1% penicillin/streptomycin, with pH 7.4. Both cells were cultured at 37 °C with 5% CO_2_.

#### 2.3.3. Cell Viability

The cells were seeded in a 96-well microplate with a density of 1.5 × 10^4^ cells per well and cultured for 24 h. The culture medium was then removed, and the cells were washed once with PBS. The washed cells were treated with BPF extract and the culture medium solutions (1:1, *v*:*v*) at different concentrations, ranging from 2.5 mg to 25 mg/mL, and the culture medium was added for a total volume of 100 µL. The plates were incubated for 24 h, 48 h and 72 h. After treatment, the medium was removed, the cells were washed with PBS, and then 100 µL of a 2% WST:medium solution was added (*v*:*v*). The cells were incubated at 37 °C with 5% CO_2_ for 90 min, and the absorbance was read in a microplate reader (Polaris, Celer, MG, Brazil) at 440 nm. Cell viability is given as percentage of viable cells using nonlinear regression. A 10% Triton X-100 solution was used as the positive control, while the negative control was the medium without extract. The lethal concentration for 50% of cultured cells (LC_50_) was obtained by using linear regression of at least three concentrations (R^2^ > 0.9) and their corresponding cell viabilities.

### 2.4. Qualitative Chemical Profile

Beetroot peel flour was extracted using ultrasound-assisted extraction in the absence of light in an ultrasonic bath (EASY 60 H, Elma Schmidbauer GmbH, Singen, Germany) with ultrapure water for 30 min at room temperature using a ratio of 1:10 (g:mL) sample:extracting solvent. The extract was then centrifuged (Heraeus Multifuge 3SR+, Thermo Scientific, Bremen, Germany) at 10,000× *g* for 10 min. The supernatant was removed and prepared for the targeted profile of betalain and its derivatives via direct infusion using a high-resolution Q-Extractive Plus Orbitrap mass spectrometer (Orbitrap-HRMS) (Thermo Fisher Scientific, Bremen, Germany) and for untargeted chemical profiling using Ultra-High-Performance Liquid Chromatography with High-Resolution Mass Spectrometry (UHPLC-HRMS).

For the targeted analysis, the samples were injected at a flow rate of 10 μL min^−1^ using a spray voltage of 3.6 kV in the positive ionization mode. The mass range evaluated was from *m*/*z* 100 to 1000 in order to discover the signals already described in the literature [11,25,26] for betalains and their derivatives. All the *m*/*z* signals within a mass accuracy of ±10 ppm were submitted to an MS^2^ analysis in order to evaluate their fragmentation profile and to confirm the identification. For the MS^2^ analysis, the collision energy varied between 10 and 25 eV. All direct infusion experiments were carried out with a capillary temperature of 380 °C, an S-lens radio frequency level of 60 (arbitrary units), the nitrogen sheath set at 10 arbitrary units, and a resolution power of 140,000 FWHM (full width at half maximum). All data obtained in the direct infusion experiments were processed using XCalibur software, version 2.2 (Thermo Fisher Scientific, Bremen, Germany).

The UHPLC-HRMS analysis followed the method described by Antonio et al. [27] for untargeted metabolomic profiles. Prior to the analysis, each extract was filtered through a 0.45 µm Polytetrafluoroethylene (PTFE)/Glass Microfiber (GMF) membrane (Whatman, Little Chalfont, UK). The UHPLC-HRMS analyses were carried out using a Dionex Ultimate 3000 UHPLC (Thermo Scientific, Bremen, Germany) connected to a Q-Exractive high-resolution spectrometer (Thermo Scientific, Bremen, Germany). Briefly, the chromatographic elution was carried out in an exploratory gradient from 0 to 100% of methanol (100%) against solvent B (0.1% formic acid in deionized water: 5 mM ammonium formate), with a flow rate of 0.4 mL min^−1^ and a gradient of 9% of solvent B min^−1^. The stationary phase was a Syncronis C18 column (2.1 × 50 mm, 100 Å–Thermofisher Scientific, Waltham, MA, USA). The HRMS was operated with an electrospray ionization source in the positive and negative ionization modes, from 100 to 900 *m*/*z*, in the data-dependent acquisition mode for MS^2^ fragmentation experiments.

The UHPLC-HRMS data were deconvoluted and deisotoped (Table 1) using the software MzMine 2 version 2.53 [25]. A putative identification of the compounds was carried out by comparing the fragmentation spectra with those on a public online database, Global Natural Products Social Molecular Networking (GNPS) [26]. For GNPS, the tool “Data Analysis” was used, with the following settings: Precursor Ion Mass Tolerance of 2.0 Da; Minimum Matched Peaks of 3; Fragment Ion Mass Tolerance of 0.5 Da; and Score Threshold of 0.7. An additional putative identification was also carried out by manually comparing the fragmentation pattern of the *m*/*z* signals present in the sample with data from the literature.

### 2.5. Statistical Analysis

The antioxidant activity and cellular assays were determined in triplicate, and the results are expressed as the mean ± standard deviation. The statistical analyses were carried out using GraphPad Prism software (GraphPad Software, version 5.00, San Diego, CA, USA), and the differences were analyzed using a two-way ANOVA or the *t*-test followed by Bonferroni’s post hoc test as required, with significance considered at the 95% confidence level (*p* < 0.05). Pearson’s correlation was used for dose- and time-dependent correlations [28], and a heatmap for the chemical profiling was prepared using DisplayR online software [29].

## 3. Results and Discussion

### 3.1. Antioxidant Activity of BPF Extracts

Beetroot is a well-recognized source of bioactive compounds, such as betalains [30]; carotenoids; phenolics; and a wide range of vitamins, including all the B complex vitamins and vitamins C, E and K [31,32]. These molecules have antioxidant potential that is well-reported on in the literature. However, some bioactive compounds in beetroot are thermally sensitive and can degrade during processing [11,33]. Thus, the BPF extracts were analyzed using different in vitro methods.

The results obtained regarding antioxidant activity demonstrated a clear difference between the extractors, so a gradient of extraction polarity was required to better observe the chemical nature of the antioxidant compounds that could be found in the plant material. The use of either ultrapure water or 50% ethanol revealed that some of the antioxidant compounds present in the BPF were lipophilic (best extracted in 50% ethanol) and that some were hydrophilic (best extracted in ultrapure water). It is worth mentioning that conventional solid–liquid extraction, using organic and/or aqueous solvents, is the main method used to extract bioactive compounds via chemical affinity and/or their removal from the matrix, with a strong influence of the concentration and molecular structure [34]. Sample preparation, solvent polarity, technique and temperature are factors that can influence the extraction and the contents of these compounds [35]. Table 2 shows a summary of the obtained results.

A higher concentration of gallic acid equivalents was found in the aqueous extract, but a stronger antioxidant response was found in the ethanolic extract in the DPPH, ABTS and FRAP assays. This difference in response can be explained by the nature of the compounds in the 50% ethanol extract, as they react better with DPPH and ABTS free radicals, in addition to reacting with iron, reducing its oxidation number in the FRAP assay [21,36]. In contrast, the ORAC assay follows a distinct principle related to the chain-breaking effect of antioxidants on peroxyl radicals [36].

In the ORAC assay, the aqueous extract exhibited almost seven times the response of the ethanolic extract, thus providing strong evidence that the bioactive compounds in this extract act on reactive oxygen species. Since cell metabolism can promote the formation of reactive oxygen species, the use of an assay that mimics this process is extremely relevant. Moreover, there is strong evidence that oxidative stress may lead to the development of cancer due to DNA damage, as well as increasing angiogenesis, which sustains the cell mass [3]. These results led to the selection of BPFw for the study of cell survival and for the chemical profile assessment.

### 3.2. Influence of BPFw on Breast Cancer Cell Survival

Two breast cancer cell lines were studied. MCF-7 cells are a representative model of breast cancer with positive responses to estrogen and progesterone, being non-invasive and little aggressive [37]. In contrast, MDA-MB-231 cells are a representative model of a triple-negative breast cancer that is not responsive to estrogen, progesterone or the epidermal growth factor, being a highly aggressive form of breast cancer with a very limited range of possible therapies [38,39].

Six concentrations of BPFw were tested with both breast cancer cell lines. Both lines presented responses due to the treatment, but the responses were different for each line. The LC50 ranged from 16.6 mg/mL to 22.6 mg/mL for the MCF-7 cell line and from 7.9 mg/mL to 20.1 mg/mL for the MDA-MB-231 cell line. The LC50 values varied according to the treatment time. Table 3 presents the BPF LC50 for each cell line and treatment.

**Figure 1:**
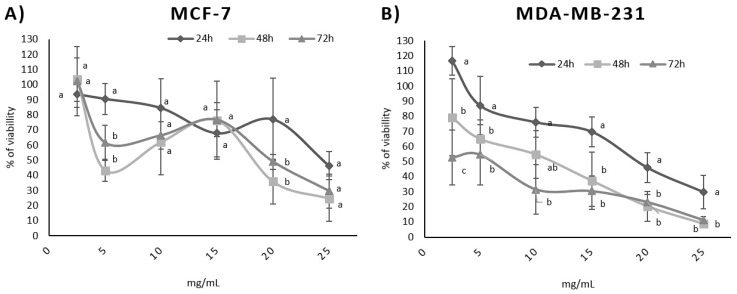
Influence of BPFw on MCF-7 (**A**) and MDA-MB-231 (**B**) breast cancer cell survival. Different letters for the same concentration indicate statistical difference between the treatment times (*p* < 0.05).

In addition, a dose-dependent effect was observed for both cell lines (Figure 1) and was confirmed via Pearson’s correlation test. A time-dependent correlation was also observed for the MDA-MB-231 cell line with a high Pearson correlation (*p* = −0.997). In contrast, the time-dependent effect was not directly proportional for the MCF-7 cell line (*p* = −0.292). Table 4 shows the *p* values for the time dependence and dose dependence tests.

Dose dependence refers to the variation in the concentration of a chemical or bioactive compound and the observed response [40]. The survival test results suggest that a higher concentration of BPF may induce less survivability for both breast cancer cell lines. This is a desirable effect, since BPF is a natural source of compounds that could be added to the diet in order to promote possible anticancer activity that may aid cancer treatment, increasing treatment efficiency [12].

Time dependence refers to the chronic consumption of the chemical or bioactive compound with the observed response, increasing with time. The MDA-MB-231 cell line presented a strong time dependence, suggesting that the chronic consumption of BPF may help to improve the response to the treatment of triple-negative breast cancer.

It has previously been reported in the literature that the compounds present in the leaves and roots of beetroot are able to act on the cell cycle of cancer cells due to the modulation of the mTOR pathway, leading to cell apoptosis [2]. Moreover, the presence of antioxidant compounds may lead to the control of the formation of reactive oxygen species, reducing angiogenesis due to a lower inflammatory state [37,41]. In addition, it has previously been demonstrated that bioactive compounds are able to act on intrinsic mechanisms of the cells, inducing apoptosis [42].

Amongst the intrinsic cell mechanisms, the mTOR pathway is crucial for the control of cell growth and metabolism. This pathway regulates a high diversity of metabolic cascades, which lead to genic expression, protein synthesis, angiogenesis maintenance and mitosis. It is a common target of cancer studies since it is highly active in this pathology, including in breast cancer [43,44]. In addition, it has also been reported that overactivation of the mTOR pathway in breast cancer cells may lead to drug resistance and apoptosis inhibition, reducing the efficiency of therapy and creating the need for the downregulation of the activity of this pathway due to molecular mechanisms [44]. Another intrinsic mechanism is due to the expression of the p53 transcriptional factor. It has previously been demonstrated that p53 is able to induce cell cycle arrest in the G1 phase, as well as being responsible for structural changes in fibroblasts leading to a reduction in cell mobility [13].

The reduction in cell survivability observed in the treatment with BPFw suggests the possible presence of bioactive compounds that can regulate the mTOR pathway and possibly induce cell apoptosis. This also explains the different results observed between the cell lines. It has previously been reported that mTOR inhibition in concomitance with endocrine therapy has a potent anti-proliferative activity against MCF-7 cells [45], suggesting synergism between the treatments. However, as triple-negative cells, MDA-MB-231 cells do not respond to hormonal treatments [38,39], leaving the mTOR pathway as a possible alternative approach to induce apoptosis in this cell line [46].

### 3.3. Chemical Profile of BPF Extracts and Their Influence on Cancer Cell Metabolism

To better understand how the bioactive compounds present in the BPFw may affect breast cancer cell survivability, untargeted chemical profiling was carried out. Thus, 9 betalains (Table 5) and 59 other compounds (Supplementary Table S1) were identified, and they are reported with descriptions of their formulae, molecular weights and structures.

In beetroot (*Beta vulgaris* L.), the main pigments are betalains [33,47]. These pigments are divided into two main groups: betacyanins, with a brilliant red-purplish color, and betaxanthins, responsible for yellow to orange colors [48]. As a product obtained from beetroot peel, the BPF presented betalains according to the chemical profile.

However, the thermal processing of the BPF promoted modifications of the betalains present in the in natura beetroot, leading to the formation of decarboxylated betalain compounds [33], which were identified via UHPLC-HRMS in this study. 15,17-bidecarboxy-betanin and 2,15-bidecarboxy-xanbetanin are thermally degraded betalain forms, both metabolites of betanin, produced via different decarboxylation and oxidation (with the liberation of H_2_O) paths, and they are precursors of 2,15,17-tridecarboxy-xanneobetanin, as proposed by Sutor-Swiezy et al. (2022) in their study of the thermal degradation of betalains [11]. In addition, another decarboxylated compound was found, and it was identified as 2,15,17-tridecarboxy-bidehydro-amaranthin [49]. As a decarboxylated compound, with missing HCOOH groups occurring on the same carbons as in 2,15,17-tridecarboxy-xanneobetanin, it is possible that the formation follows a pathway the same as or similar to the pathway presented by Sutor-Swiezy et al. (2022), with the difference of starting from amaranthin instead of betanin [11]. The BPF processing temperature was 70 °C, much higher than the temperature tolerated by the betalains, which is reported as being below 45 °C [50]. Thus, the presence of these compounds in the BPFw was expected, and this supports the thermal degradation pathways reported in the literature [11,49].

As aforementioned, anticancer activity has previously been reported for beetroot. However, most of these were reports of in natura extracts [2,6,7] or products made with beetroot flesh [51], air-dried beetroot [7] or freeze-dried beetroot peels [10], and it is well-known that the thermal processing of the flour can degrade bioactive compounds [16].

**Table 5:** Identification of betalains in the BPFw by direct injection into the Orbitrap-HRMS system.

Compound	Formula	[M + H]^+^ Theoretical	[M + H]^+^ Observed	Mass Accuracy (ppm)	Molecular Structure	Reference
Betacyanins						
Betanin	C_24_H_26_N_2_O_13_	551.1508	551.1500	−1.38	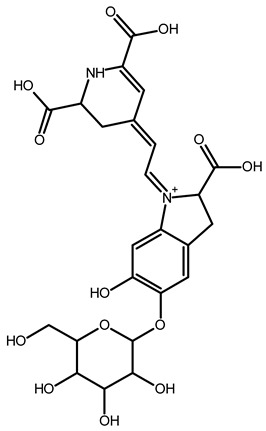	[49]
15,17-bidecarboxy-betanin	C_16_H_17_N_2_O_4_	301.1194	301.1185	0.72	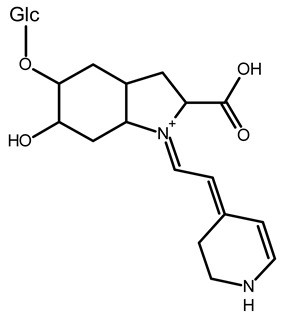	[11]
2,15-bidecarboxy-xanbetanin	C_16_H_15_N_2_O_4_	461.1547	461.1544	−2.99	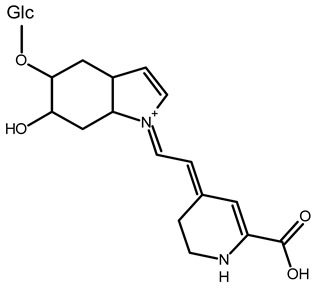	[11]
2,15,17-tridecarboxybidehydro-amaranthin	C_27_H_30_N_2_O_13_	591.1821	591.1832	1.86	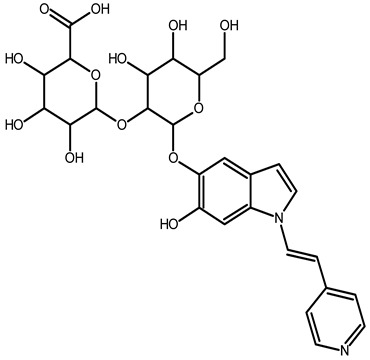	[49]
6′-feruloyl-betanin	C_34_H_35_N_2_O_16_^+^	727.1981	727.1962	−2.61	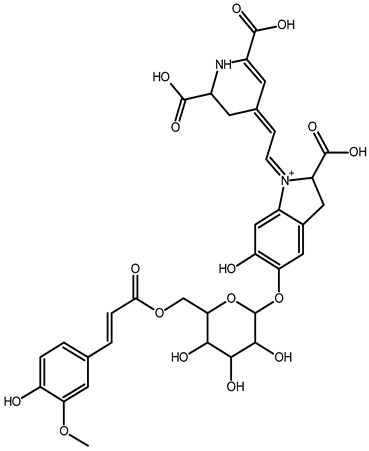	[52]
Betaxanthins						
Ethanolamine-betaxanthin	C_12_H_14_N_2_O_5_	255.0970	255.0988	7.05	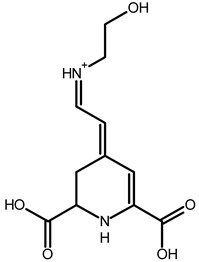	[49,53]
Threonine-betaxanthin	C_13_H_16_N_2_O_7_	313.1022	313.1027	1.59	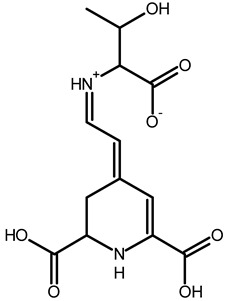	[49,53]
Vulgaxanthin I (glutamine-betaxanthin)	C_14_H_17_N3_87_	340.1134	340.1135	0.29	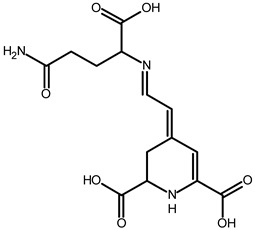	[49]
Vulgaxanthin IV (leucine-betaxanthin)	C_15_H_20_N_2_O_6_	325.1387	325.1389	0.61	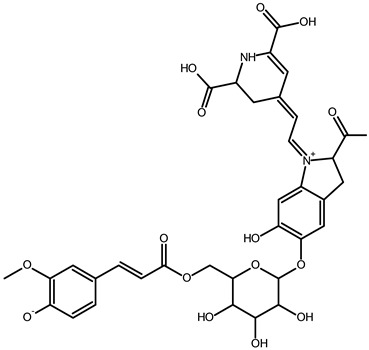	[49]

Regardless of the identified thermal degradation of betalains, the BPFw still presented anticancer activity. In general, betalains have been reported as biomolecules that are able to interact with the mTOR pathway by lowering *p*-PI3K and *p*-AKT levels in lung cancer cells [6] and modulating mTOR levels in prostate cancer cells [2]. The activation of the caspase pathway and a reduction in angiogenesis have also been observed [54]. Of the betacyanins identified, betanin has previously been reported as an anticancer agent due to acceleration of DNA repair in Caco-2 colorectal cancer cells and DNA protection against oxidative damage in neutrophils [55]. Betaxanthins have also been reported as anticancer agents [56], but information about specific molecules and their anticancer mechanisms is scarce. However, the literature demonstrates that indicaxanthin is involved in the chemoprotection of the DNA in colorectal cancer models [57]. Unfortunately, indicaxanthin was not identified in the BPFw in the present chemical profiling study.

The metabolic influences reported for betalains are desirable in the treatment of cancer due to the modulation of molecular pathways that lead to cell death via apoptosis, since, in cancer, apoptotic mechanisms are usually inefficient or evaded by the cell, leading to uncontrolled tissue development [58].

**Figure 2:**
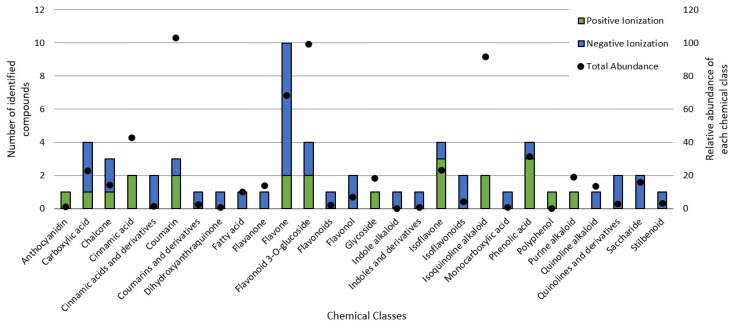
Chemical classes found in the BPFw in both the positive and negative ionization modules.

**Figure 3:**
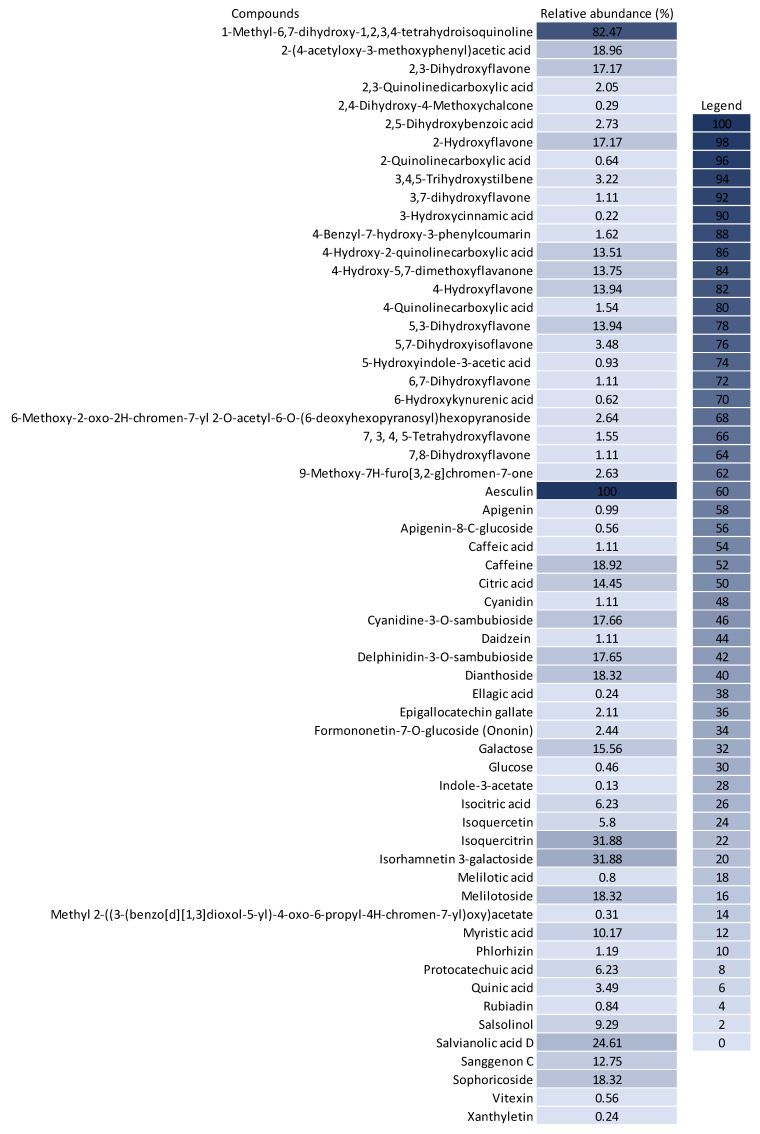
Relative abundance (%) of the compounds identified in the BPFw as compared to the abundance of aesculin (100%).

The untargeted chemical profile analysis enabled the identification of 59 compounds (Supplementary Materials) from different chemical classes, such as carboxylic acids, chalcones, coumarins, flavones, glycosylated flavonoids, isoflavones and phenolic acids. Flavonoids were the largest group occurring in the UHPLC-HRMS analysis, of which, most of the compounds identified were flavones, with ten different structures identified. Considering the relative abundance of the compounds identified within the sample, the coumarins were the second most abundant, and aesculin was the most abundant single compound in the sample. Figure 2 shows the distribution of the chemical classes found in each ionization mode, the number of compounds and the relative abundance of each chemical class. In addition, Figure 3 shows the relative abundance of the compounds identified as compared to aesculin, which was the most abundant single compound.

Flavonoids are an identified group receiving special interest in cancer therapy [4], and they were the most abundant group of compounds, including all their derivatives. Glycosylated flavonoids were the second most abundant group in the BPFw, with four different components identified: Cyanidine-3-o-sambubioside, Delphinidin-3-o-sambubioside, Isoquercitrin and Isorhamnetin 3-galactoside. Other derivatives were identified and grouped accordingly, namely, flavones, flavonones, flavonols, isoflavones, non-glycosylated flavonoids and anthocyanidins. All these compounds present a main polyphenolic structure (A and C rings) with a B ring attached at carbon 2 (flavonoids) or carbon 3 (isoflavonoids) of the C ring, and they could have other compounds attached, leading to a variety of possible classifications [4]. Flavonoids are poorly soluble in water due to their highly saturated polyphenolic structure. However, the abundance of glycosylated flavonoids being greater than that of the other flavonoid classes was expected due to the presence of the attached saccharide, which contributes positively to the hydro-solubility of these compounds [59]. This large group of compounds has been reported as anticancer agents due to their different mechanisms, including the apoptosis induced by reticulum endoplasmic stress and/or by reactive oxygen species stress [4,58], gene [60].

Within the class of coumarins, the literature presents evidence that aesculin displays highly relevant anticancer activity. Kaneko et al. (2007) demonstrated that the administration of aesculin to male rats promoted better DNA protection against chemically induced colorectal cancer [61]. In addition, it was demonstrated by Rashmi et al. (2020) that aesculin has a relevant effect on MDA-MB-231 breast cancer cells [62]. It has also been previously reported that aesculin can be converted into esculetin in human cells, with esculetin promoting a protective effect against breast cancer formation [61].

The third most abundant group of compounds was the group of isoquinoline alkaloids. Only salsolinol was identified in this class, but it was the second most abundant individual compound. This compound has been reported as an inhibitor of the mitochondrial complex II, leading to the downregulation of energetic metabolism in neuroblastoma cell models [63]. In addition, salsolinol has recently been reported as a neuroprotective agent against reactive oxygen species and induced necrosis [64]. Although not yet described specifically for salsolinol, the class of isoquinoline alkaloids has already been reported as capable of inhibiting the activity of telomerase, an enzyme involved in DNA modulation and in the mechanism of avoiding apoptosis, which leads to cell death [65]. Isoquinoline alkaloids have also been reported to bind to microtubes during the cell cycle, leading to cycle arrest followed by apoptosis in MCF-7 cancer cells [66].

Cinnamic acids are another group of interest for cancer treatment and prevention. In the BPFw, four compounds were found: melilotoside, salvianolic acid D, 3-hydroxycinnamic acid and caffeic acid. Of these, melilotose was the most abundant, followed by salvianolic acid D, with this molecule being a dimer of caffeic acid [67]. Caffeic acid has previously been reported as a potential modulator of cancer pathways, specifically modulating autophagy and apoptotic pathways [68]. Pelinson et al. (2019) studied the effect of caffeic acid on the metabolism and gene expression of melanoma cancer cells. The authors found that caffeic acid was able to promote cell death and modulate the cell cycle while increasing the gene expressions of caspases, indicating a positive modulation of apoptosis [69]. In breast cancer, it has been demonstrated that both caffeic acid and caffeine (also identified in the BPFw) are able to suppress the expressions of estrogen receptors in MCF-7 cells and that caffeine can reduce the expressions of insulin-like receptors in MCF-7 and MDA-MB-231 cells, leading to cell death [70].

The diversity of compounds identified in the BPFw and the different anticancer mechanisms reported for these compounds suggest that the death of breast cancer cells observed in our cell assays could be due to a synergism of these compounds, possibly acting via different pathways in a complementary way, amplifying the cytotoxic effect on the cell types studied. This synergism has previously been evidenced by the reported chemo-sensitization promoted by betacyanins in the anticancer activity of vitexin and doxorubicin in different cancer models [71]. Considering that vitexin was identified in the BPFw and that beetroot is a natural source of betacyanins, it is suggested that the synergistic effect of these molecules occurred in the BPFw.

In addition, regardless of the molecular relaxation of synergism between the identified compounds, the synergism of the anticancer action may occur due to the parallel and concomitant activation of different pathways that lead to the same metabolic result, reinforcing its activity and leading to cell death.

The discovery of natural sources of bioactive compounds with anticancer activities is essential for cancer treatment due to the potential reduction in the side effects promoted by conventional therapies [72]. Thus, the present study provides strong evidence that beetroot peel flour could be a valuable ally in breast cancer treatment as a source of anticancer compounds.

## 4. Conclusions

The beetroot peel flour presented satisfactory in vitro antioxidant activity, with strong evidence of action on reactive oxygen species. The cell assays demonstrated that the BPFw had a great influence on triple-negative breast cancer cells (MDA-MB-231), reducing their viability in a dose- and time-dependent manner. In contrast, the viability of the MCF-7 cells (positive for estrogen receptor) did not decrease in a time-dependent manner. However, the viability of these cells did present dose-dependent behavior. The chemical profiling of the BPFw demonstrated the presence of nine betalains, including five betacyanins (betanin and four derivatives) and four betaxanthins. In addition, aesculin was shown to be the most abundant compound, with another 58 compounds being identified. Of these compounds, flavonoids and their derivatives, isoquinoline alkaloids and cinnamic acids were the chemical classes showing strong evidence of anticancer activity via different pathways, supporting the results obtained in the cell assays. Finally, the results demonstrate that, regardless of the thermal degradation promoted by the processing of the peel to obtain the flour, the BPF still showed relevant antioxidant and anticancer activities, supporting the production of vegetable flours from vegetable byproducts.

## Figures and Tables

**Table 1:** Data mining steps used in MzMine 2 v. 2.53 for the deconvolution of the UHPLC-HRMS data.

Ionization Mode	Positive	Negative
Step 1. Mass detection		
Mass detector	Exact mass	
MS level	1	
Noise level	4.00 × 10^6^	5.00 × 10^5^
MS level	2	
Noise level	1.00 × 10^4^	1.00 × 10^4^
Step 2. ADAP chromatogram builder		
Min group size of scan	5	
Group intensity threshold	1.20 × 10^7^	1.00 × 10^4^
Min highest intensity	6.00 × 10^4^	1.00 × 10^4^
*m*/*z* tolerance	0.0 Da or 10 ppm	
Step 3. Chromatogram deconvolution		
Algorithm	baseline cut-off	
Min. peak height	1.00 × 10^6^	1.00 × 10^5^
Peak duration (min)	0 to 4	
Baseline level	1.20 × 10^5^	4.00 × 10^5^
*m*/*z* range for MS2 scan pairing (Da)	0.01	
*m*/*z* range for MS2 scan pairing (min)	0.2	
*m*/*z* center calculation	Average	
Step 4. Deisotope		
*m*/*z* tolerance	0.0 Da or 10 ppm	
Retention time tolerance (%)	30	
Maximum charge	3	
Representative isotope	most intense	

**Table 2:** Summary of the in vitro antioxidant activity in the beetroot peel flour extracts.

Extract	In Vitro Analysis				
	TPC (mg GAE/100 g)	DPPH (µmol T/100 g)	ABTS (µmol T/100 g)	FRAP (µM FS/g)	ORAC (µmol T/100 g)
BPFw	301.64 ± 0.20 a	259.72 ± 17.26 a	7692.31 ± 0.01 a	29.72 ± 0.04 a	3032.78 ± 55.00 a
BPFe	246.99 ± 0.03 b	299.37 ± 54.71 b	9081.50 ± 0.03 b	96.98 ± 0.03 b	462.50 ± 89.00 b

Different letters in the same column indicate a statistical difference between the values (*p* < 0.05).

**Table 3:** Lethal concentration values (LC50) of BPFw for the breast cancer cell lines in in vitro assays.

Treatment Time	LC50 (mg/mL)	
	MCF-7	MDA-MB-231
24 h	22.57 ± 3.12 a*	20.12 ± 3.42 a
48 h	16.64 ± 6.72 b*	13.21 ± 1.90 b
72 h	20.81 ± 3.53 a*	7.87 ± 7.13 c

LC50 values obtained from a linear regression of at least three concentrations for each assay (R^2^ > 0.9) from three independent experiments. Different lowercase letters in the same column indicate statistical difference according to time exposure (*p* < 0.05). Signalized data (*) in the same row indicate statistical difference between the cell lines (*p* < 0.05).

**Table 4:** Dose and time dependence of the anticancer effect of BPFw according to Pearson’s correlation.

Cell Line	Dose-Dependent Correlation for Each Exposure Time (*p* Values)			Time-Dependent Correlation (*p* Values)
	24 h	48 h	72 h	
MCF-7	−0.904	−0.700	−0.819	−0.292
MDA-MB-231	−0.969	−0.996	−0.968	−0.997

*p* values = −1 means a perfect negative correlation between the variables. *p* values = 1 means a perfect positive correlation between the variables. *p* values = 0 means no correlation between the variables.

## Data Availability

The data presented in this study are available in article and Supplementary Materials.

## Supplementary Materials

The following supporting information can be downloaded at: https://www.mdpi.com/article/10.3390/metabo13020277/s1, Table S1: Compounds identified in the BPF extract by UHPLC-HRMS using FBMN-GNPS workflow.
