# Supplementary material for: Antioxidant Capacity, Antitumor Activity and Metabolomic Profile of a Beetroot Peel Flour
Source: Metabolites. 2023 Feb 14;13(2):277. doi: 10.3390/metabo13020277 (PMC9961284; doi:10.3390/metabo13020277)
Supplement: Supplementary file 1 [file metabolites-13-00277-s001.zip › metabolites-2087974-supplementary.pdf]

Supplementary Table S1: Compounds identified in the BPF extract by UHPLC-HRMS using FBMN-GNPS workflow.

Compound	Ionization module	Chemical Class	Formula	Molecular Weight (g/mol)	MQScore	Molecular structure
2-(4-acetyloxy-3-methoxyphenyl)acetic acid	Positive	Phenolic acid	$C_{11}H_{12}O_5$	224.21	0.744272	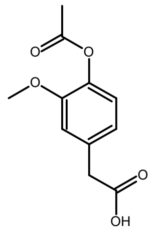
2,3-dihydroxyflavone	Positive	Flavone	$C_{15}H_{10}O_4$	254.24	0.909874	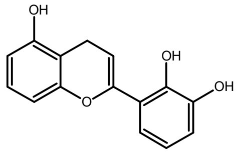
2,3-quinolinedicarboxylic acid	Negative	Quinolines and derivatives	$C_{11}H_7NO_4$	217.18	0.809719	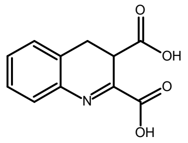
2,4-dihydroxy-4-methoxychalcone	Negative	Chalcone	$C_{16}H_{14}O_4$	270.28	0.727163	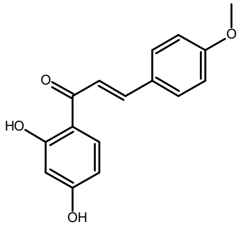
2,5-dihydroxybenzoic acid	Negative	Phenolic acid	$C_7H_6O_4$	154.12	0.937293	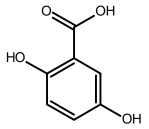
2-hydroxyflavone	Positive	Flavone	$C_{15}H_{10}O_3$	238.24	0.920667	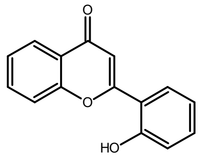
2-quinolinecarboxylic acid	Positive	Carboxylic acid	$C_{10}H_7NO_2$	173.17	0.774372	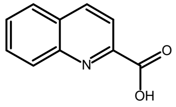

Compound	Ionization module	Chemical Class	Formula	Molecular Weight (g/mol)	MQScore	Molecular structure
3,4,5-trihydroxystilbene	Negative	stilbenoid	$C_{14}H_{12}O_3$	228.24	0.729505	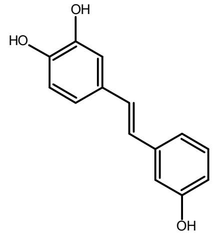
3,7-dihydroxyflavone	Negative	flavonol	$C_{15}H_{10}O_4$	254.24	0.816893	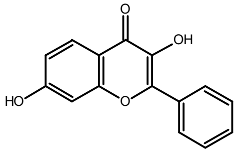
3-hydroxycinnamic acid	Negative	Cinnamic acids and derivatives	$C_9H_8O_3$	164.16	0.727908	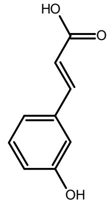
4-benzyl-7-hydroxy-3-phenylcoumarin	Negative	Isoflavonoids	$C_{22}H_{16}O_3$	328.4	0.762824	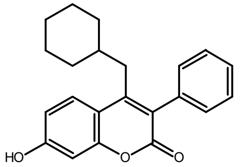
4-hydroxy-2-quinolinecarboxylic acid	Negative	quinoline alkaloid	$C_{10}H_7NO_3$	189.17	0.893112	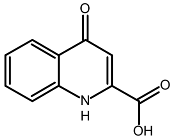
4-hydroxy-5,7-dimethoxyflavanone	Negative	flavanone	$C_{17}H_{16}O_5$	300.3	0.905707	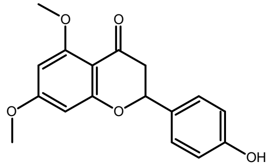
4-hydroxyflavone	Negative	flavone	$C_{15}H_{10}O_3$	238.24	0.897316	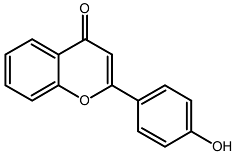

Compound	Ionization module	Chemical Class	Formula	Molecular Weight (g/mol)	MQScore	Molecular structure
4-quinolinecarboxylic acid	Negative	carboxylic acid	$C_{10}H_7NO_2$	173.17	0.92021	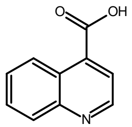
5,3-dihydroxyflavone	Negative	flavone	$C_{15}H_{10}O_4$	254.24	0.943867	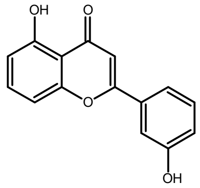
5,7-dihydroxyisoflavone	Positive	isoflavone	$C_{15}H_{10}O_4$	254.24	0.804587	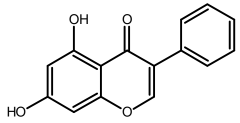
5-Hydroxyindole-3-acetic acid	Negative	Indoles and derivatives	$C_{10}H_9NO_3$	191.18	0.750671	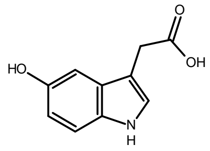
6,7-dihydroxyflavone	Negative	flavone	$C_{15}H_{10}O_4$	254.24	0.789647	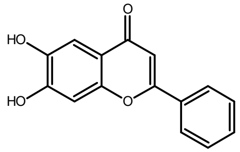
6-hydroxykynurenic acid	Negative	Quinolines and derivatives	$C_{10}H_7NO_4$	205.17	0.72652	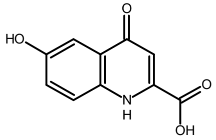
6-methoxy-2-oxo-2h-chromen-7-yl 2-o-acetyl-6-o-(6-deoxyhexopyranosyl)hexopyranoside	Negative	Coumarins and derivatives	$C_{24}H_{30}O_{14}$	542.5	0.796464	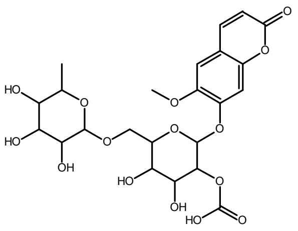

Compound	Ionization module	Chemical Class	Formula	Molecular Weight (g/mol)	MQScore	Molecular structure
7, 3, 4, 5-tetrahydroxyflavone	Negative	flavone	$C_{15}H_{10}O_6$	286.24	0.797703	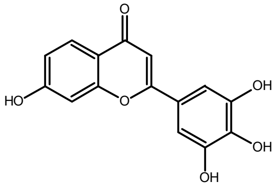
7,8-dihydroxyflavone	Negative	flavone	$C_{15}H_{10}O_4$	254.24	0.798348	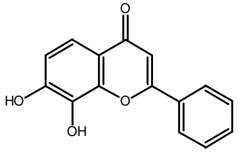
9-methoxy-7h-furo[3,2-g]chromen-7-one	Positive	coumarin	$C_{12}H_8O_4$	216.19	0.718438	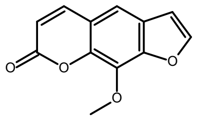
Aesculin	Negative	coumarin	$C_{15}H_{16}O_9$	340.28	0.915798	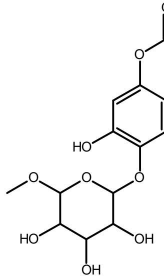
Apigenin	Negative	flavone	$C_{15}H_{10}O_5$	270.24	0.81928	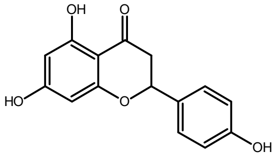
Apigenin-8-c-glucoside	Negative	flavone	$C_{21}H_{20}O_{10}$	432.4	0.922017	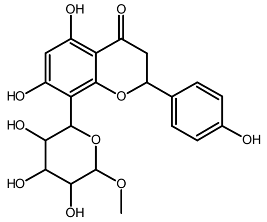

Compound	Ionization module	Chemical Class	Formula	Molecular Weight (g/mol)	MQScore	Molecular structure
Caffeic acid	Negative	Cinnamic acids and derivatives	$C_9H_8O_4$	180.16	0.775537	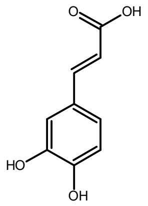
Caffeine	Positive	purine alkaloid	$C_8H_{10}N_4O_2$	194.19	0.942158	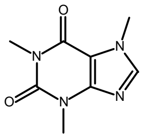
Citric acid	Negative	carboxylic acid	$C_6H_8O_7$	192.12	0.994374	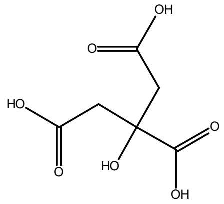
Cyanidin	Positive	anthocyanidin	$C_{15}H_{11}O_6^+$	287.24	0.89836	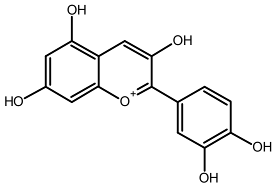
Cyanidine-3-o-sambubioside	Negative	flavonoid 3-O-glucoside	$C_{26}H_{29}O_{15}^+$	581.5	0.745008	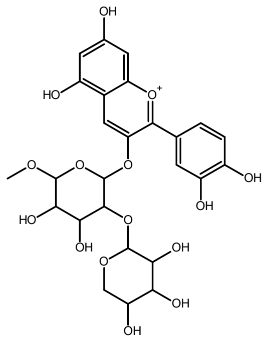
Daidzein	Negative	isoflavone	$C_{15}H_{10}O_4$	254.24	0.809828	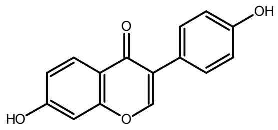

Compound	Ionization module	Chemical Class	Formula	Molecular Weight (g/mol)	MQScore	Molecular structure
Delphinidin-3-o-sambubioside	Negative	flavonoid 3-O-glucoside	$C_{26}H_{29}O_{16}^{+}$	597.5	0.750745	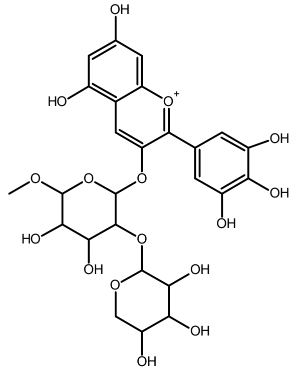
Dianthoside	Positive	glycoside	$C_{12}H_{16}O_{8}$	288.25	0.902466	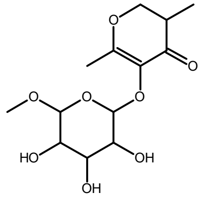
Ellagic acid	Positive	polyphenol	$C_{14}H_{6}O_{8}$	302.19	0.770144	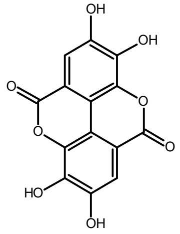
Epigallocatechin gallate	Negative	Flavonoids	$C_{22}H_{18}O_{11}$	458.4	0.753114	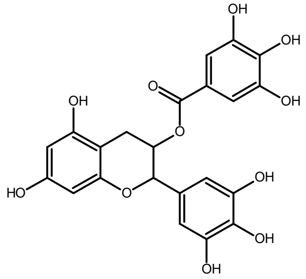
Formononetin-7-o-glucoside	Negative	Isoflavonoids	$C_{22}H_{22}O_{9}$	430.4	0.865509	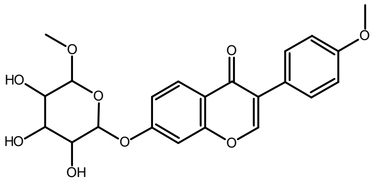

Compound	Ionization module	Chemical Class	Formula	Molecular Weight (g/mol)	MQScore	Molecular structure
Galactose	Negative	saccharide	$C_6H_{12}O_6$	180.16	0.976578	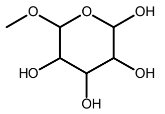
Glucose	Negative	saccharide	$C_6H_{12}O_6$	180.16	0.808242	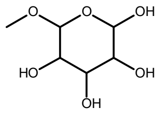
Indole-3-acetate	Negative	indole alkaloid	$C_{10}H_8NO_2^-$	174.18	0.965575	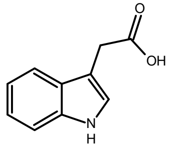
Isocitric acid	Negative	carboxylic acid	$C_6H_8O_7$	192.12	0.80607	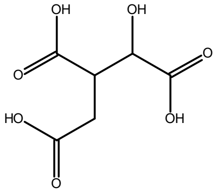
Isoquercetin	Negative	flavonol	$C_{21}H_{20}O_{12}$	464.4	0.728476	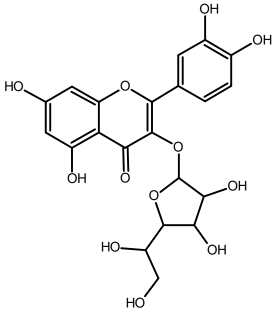
Isoquercitrin	Positive	flavonoid 3-O-glucoside	$C_{21}H_{20}O_{12}$	464.4	0.946136	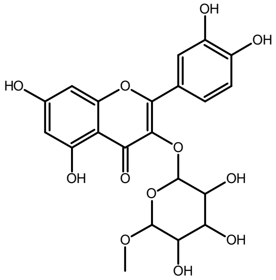

Compound	Ionization module	Chemical Class	Formula	Molecular Weight (g/mol)	MQScore	Molecular structure
Isorhamnetin 3-galactoside	Positive	flavonoid 3-O-glucoside	$C_{22}H_{22}O_{12}$	478.4	0.954748	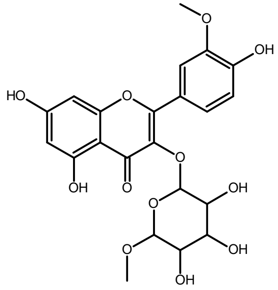
Melilotic acid	Negative	monocarboxylic acid	$C_9H_{10}O_3$	166.17	0.813388	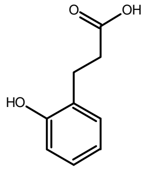
Melilotoside	Positive	cinnamic acid	$C_{15}H_{18}O_8$	326.3	0.926209	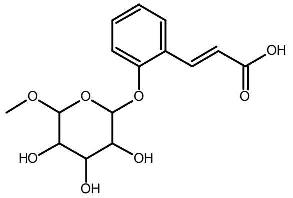
Methyl 2-((3-(benzo[d][1,3]dioxol-5-yl)-4-oxo-6-propyl-4h-chromen-7-yl)oxy)acetate	Positive	isoflavone	$C_{22}H_{20}O_7$	396.4	0.719086	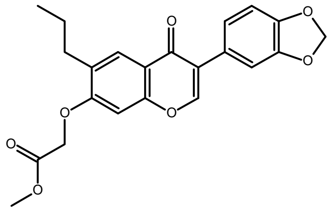
Myristic acid	Negative	fat acid	$C_{14}H_{28}O_2$	228.37	0.854644	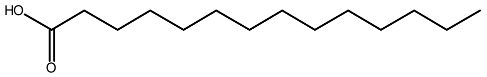
Phlorhizin	Negative	chalcone	$C_{21}H_{24}O_{10}$	436.4	0.803055	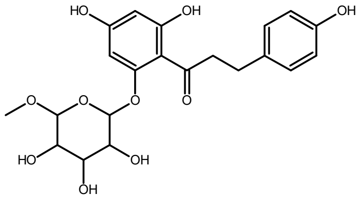

Compound	Ionization module	Chemical Class	Formula	Molecular Weight (g/mol)	MQScore	Molecular structure
Protocatechuic acid	Positive	phenolic acid	$C_7H_6O_4$	154.12	0.809345	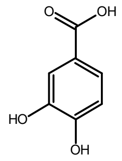
Quinic acid	Positive	phenolic acid	$C_7H_{12}O_6$	192.17	0.803206	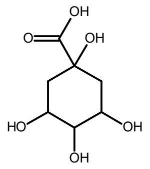
Rubiadin	Negative	dihydroxyanthraquinone	$C_{15}H_{10}O_4$	254.24	0.90528	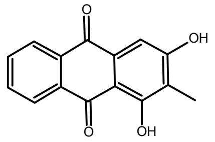
Salsolinol	Positive	isoquinoline alkaloid	$C_{10}H_{13}NO_2$	179.22	0.844646	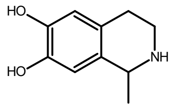
Salvianolic acid D	Positive	cinnamic acid	$C_{20}H_{18}O_{10}$	418.3	0.812422	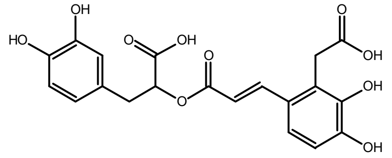
Sanggenon C	Positive	chalcone	$C_{40}H_{36}O_{12}$	708.7	0.918472	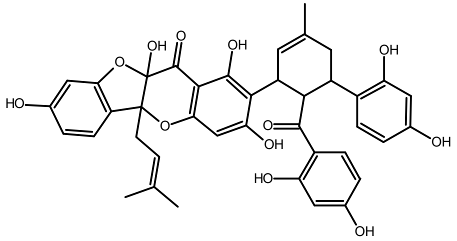

Compound	Ionization module	Chemical Class	Formula	Molecular Weight (g/mol)	MQScore	Molecular structure
Sophoricoside	Positive	isoflavone	$C_{21}H_{20}O_{10}$	432.4	0.903133	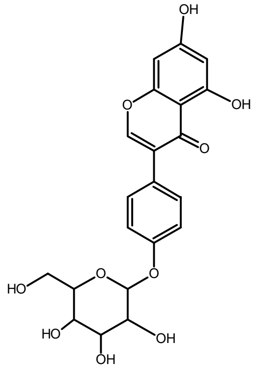
Vitexin	Negative	flavone	$C_{21}H_{20}O_{10}$	432.4	0.928021	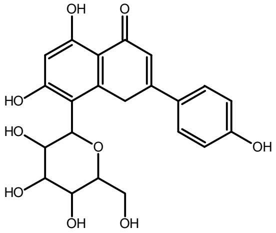
Xanthyletin	Positive	coumarin	$C_{14}H_{12}O_3$	228.24	0.815527	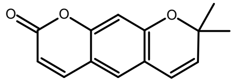
